# New Avian Paramyxoviruses Type I Strains Identified in Africa Provide New Outcomes for Phylogeny Reconstruction and Genotype Classification

**DOI:** 10.1371/journal.pone.0076413

**Published:** 2013-10-18

**Authors:** Renata Servan de Almeida, Saliha Hammoumi, Patricia Gil, François-Xavier Briand, Sophie Molia, Nicolas Gaidet, Julien Cappelle, Véronique Chevalier, Gilles Balança, Abdallah Traoré, Colette Grillet, Olivier Fridolin Maminiaina, Samia Guendouz, Marthin Dakouo, Kassim Samaké, Ould El Mamy Bezeid, Abbas Diarra, Hassen Chaka, Flavie Goutard, Peter Thompson, Dominique Martinez, Véronique Jestin, Emmanuel Albina

**Affiliations:** 1 CIRAD, UMR CMAEE, Montpellier, France; 2 INRA, UMR1309 CMAEE, Montpellier, France; 3 Anses-Ploufragan-Plouzané, VIPAC, French Reference Laboratory for Avian Influenza and Newcastle Disease, Ploufragan, France; 4 CIRAD, UPR AGIRS, Montpellier, France; 5 Laboratoire Central Vétérinaire, Bamako, Mali; 6 FOFIFA-DRZV, Antananarivo, Madagascar; 7 Centre National d’Elevage et de Recherche Vétérinaires (CNERV), Nouakchott, Mauritania; 8 National Animal Health Diagnostic and Investigation Center (NAHDIC), Sebeta, Ethiopia; 9 Faculty of Veterinary Science, University of Pretoria, Pretoria, South Africa; 10 CIRAD, UMR CMAEE, Petit-Bourg, Guadeloupe, France; Institut Pasteur of Shanghai, Chinese Academy of Sciences, China

## Abstract

Newcastle disease (ND) is one of the most lethal diseases of poultry worldwide. It is caused by an avian paramyxovirus 1 that has high genomic diversity. In the framework of an international surveillance program launched in 2007, several thousand samples from domestic and wild birds in Africa were collected and analyzed. ND viruses (NDV) were detected and isolated in apparently healthy fowls and wild birds. However, two thirds of the isolates collected in this study were classified as virulent strains of NDV based on the molecular analysis of the fusion protein and experimental *in vivo* challenges with two representative isolates. Phylogenetic analysis based on the F and HN genes showed that isolates recovered from poultry in Mali and Ethiopia form new groups, herein proposed as genotypes XIV and sub-genotype VIf with reference to the new nomenclature described by Diel’s group. In Madagascar, the circulation of NDV strains of genotype XI, originally reported elsewhere, is also confirmed. Full genome sequencing of five African isolates was generated and an extensive phylogeny reconstruction was carried out based on the nucleotide sequences. The evolutionary distances between groups and the specific amino acid signatures of each cluster allowed us to refine the genotype nomenclature.

## Introduction

Newcastle disease (ND) is one of the most severe infectious diseases of birds, particularly poultry, and has been the cause of major economic losses worldwide [1]. It is one of the 14 avian diseases notifiable to the World Organization for Animal Health (Office International des Epizooties, OIE) [2]. The cause of ND, Newcastle disease virus (NDV) or avian paramyxovirus type 1 (APMV-1), belongs to the *Avulavirus* genus, *Paramyxoviridae* family, and has a negative-sense single-stranded RNA genome of about 15.2 kilobases. The genome encodes eight proteins, nucleocapsid (NP), phosphoprotein (P), matrix (M), fusion (F), hemagglutinin-neuraminidase (HN), a large RNA-directed RNA polymerase (L), and two additional nonstructural proteins, V and W, generated by RNA editing during P gene transcription [3], [4]. NDV can be categorized into highly pathogenic (velogenic), intermediate (mesogenic), and nonpathogenic (lentogenic) strains based on pathogenicity in chickens [5]. Although V, HN, NP, P and L proteins play a role in virulence [6], [7], [8], [9], the most important molecular determinant of virulence appears linked to the amino acid motif present at the protease cleavage site of the F0 precursor of the fusion protein [10]. In virulent isolates, this motif is constituted of basic amino acids, and rapid typing of this region by RT-PCR and sequencing is a good indicator of the NDV pathotype. However, other viral factors affect the virulence of isolates, so pathogenicity should be confirmed by *in vivo* tests, including the intracerebral pathogenicity index (ICPI) in 1-day-old chickens, the mean death time (MDT) of specific-pathogen-free hen’s embryos after inoculation, and the intravenous pathogenicity index (IVPI) in 6-week-old chickens [2].

NDV strains are divided into two clades (class I and class II) according to the genome size and the sequence of the F and L genes [4]. Restriction enzyme site mapping of the F protein gene and phylogenetic analysis of the partial nucleotide sequence of the F gene have been used to classify NDV of class II [11], [12]. However, there is no consensus on NDV classification and taxonomy, since some authors use the classification of the group of Lomniczi and Ballagi-Pordany [11], [12] based on “genotypes” whereas others use the “lineage” classification of Aldous et al [1]. Both cover distinct isolate clusters but are based on the same genomic information. According to the evolutionary distances, Miller et al [13] showed inconsistencies between the two nomenclatures (for example lineage 3 is not monophyletic and contains genotypes III, IV, V, and VIII: detailed discrepancies between the two nomenclatures can be found in Table S1). Calling for objective criteria to unify the NDV nomenclature, those authors favored the use of genotypes. In the first genotype classification [12], genotype I contained mostly avirulent strains whereas genotypes II, III, and IV were involved in the first panzootic that started in 1920 and vanished around 1950. Genotypes V, VIa, and VIII were responsible for the second panzootic between the 1960s and the 1970s. Sub-genotypes VIb, VIc, and VId caused the third panzootic that emerged from pigeons during the 1980s, and sub-genotypes VIIa, VIIb, VIIc, and VIId appeared in the 1980s and the 1990s in the Far East, Europe, and South Africa [14], [15]. Genotype VII has been the predominant genotype circulating throughout the world, particularly in Asia and Africa, and it was recently reported in South America [16], [17], [18]. South African, European, American, and Asian isolates were previously typed as genotypes VIb, VIIb, and VIII [12], [16], [17], [19], [20]. In Uganda, strains from an undetermined genotype but close to genotype VI were also isolated [21]. In many parts of Africa, ND is considered endemic but only few data are available on virus isolates circulating there. In West Africa, new sub-genotypes VIIf, VIIg, and VIIh were recently described [22], [23]. Based on phylogenetic analyses of a partial F coding sequence of NDV isolates recovered from apparently healthy poultry in Mali in 2007 and 2008, we previously proposed a new sub-genotype, VIIi [24]. In Madagascar, other original strains were also isolated, sequenced, and ascribed to a new genotype, genotype XI [24], [25]. Six new genotypes were subsequently proposed by others, with objective criteria, to unify the genotype nomenclature of NDV [26], [27]. In this work, new African NDV isolates are described, and their sequences and others publicly available were included in an extensive phylogeny reconstruction based on various methods. It is shown that the “genotype” nomenclature is better adapted to the resulting genetic discrimination of NDV isolates. In addition, 10 sub-genotypes are defined. According to these results and previous publications, a rooted classification with 14 distinct genotypes is now proposed.

## Materials and Methods

### Ethics Statement

All animal experiments (ICPI tests) were conducted according to internationally approved OIE standards, under authorizations set forth by the director of the veterinary services of Côtes d’Armor on behalf of the prefect of Côtes d’Armor (N°22–18) and by the director of the veterinary services of Hérault on behalf of the prefect of Hérault (N° 34–114). The experiments were approved by the Regional Committee for Ethics in animal experiments (Comité d’éthique ComEth Afssa/ENVA/UPEC) under number 14/06/2011-4. Certificates are available from the authors upon request.

### Samples

Samples were collected in the framework of the Gripavi project (http://gripavi.cirad.fr/en/) launched in 2007 and terminated in 2011 by CIRAD in collaboration with the *Laboratoire Central Vétérinaire* of (LCV) Bamako (Mali), *Centre National d’Elevage et de Recherche Veterinaire* (CNERV) of Nouakchott (Mauritania), the *Département de Recherche Zootechnique et Vétérinaire du Centre National de la Recherche Appliquée au Développement Rural* (FOFIFA-DRZV) in Madagascar, the *National Animal Health Diagnostic and Investigation Center* (NADHIC) of Sebeta in Ethiopia, and with the support of the French Ministry of Foreign Affairs. Cloacal and tracheal swabs were collected from 9,609 domestic and 10,343 wild birds in different areas of Mali, Mauritania, Madagascar, and Ethiopia from 2007 to the end of 2011. The samples were dipped in a transport medium consisting of isotonic phosphate-buffered saline, pH 7.0–7.4, with antimicrobial additives (penicillin 10,000 U/mL, streptomycin 10 mg/mL, amphotericin B 25 µg/mL, and gentamycin 250 µg/mL) supplemented with 20% glycerol and stored in liquid nitrogen containers. They were shipped in dry ice and kept in the laboratory at −80°C until processing. All samples were manipulated in a bio-safety level 3 laboratory.

### RNA Extraction and Molecular Detection

Viral RNA was extracted from samples by a high throughput automated workstation Biomek FX^P^ (Beckman) using the Nucleospin RNA virus kit (Macherey Nagel). The viral RNA was resuspended in nuclease-free water and stored at −80°C. NDV was detected on the F gene by real-time one step RT-PCR (rRT-PCR) using Stratagene Machine Mx3000 or 3005. The forward primer was F+4839 5′-TCCGGAGGATACAAGGGTCT [28] and the reverse primer was F2AS 5′-TGGCAGCATTCTGGTTGGCT [29]. RT-PCR was carried out in a 25 µl reaction mixture with the Brilliant SYBR Green QRT-PCR II Master mix Kit (Stratagene) according to the manufacturer’s instructions. The conditions for reverse transcription were 50°C for 30 min and 95°C for 15 min. PCR consisted of 40 cycles of 95°C for 30 sec, 60°C for 1 min, and 72°C for 30 sec, followed by the final dissociation stage: 1 min 95°C, 30 sec 55°C, 30 sec 95°C.

### Virus Isolation and Pathogenicity Tests

All samples positive on rRT-PCR were inoculated into the allantoic cavities of 9- to 11-day-old embryonated fowl eggs from a commercial specific-pathogen-free flock. After one to three passages, allantoic liquid from dead eggs was tested by NDV rRT-PCR, and NDV-positive samples were then stored at −80°C and used as working stock for sequence analysis.

For the determination of pathogenicity, the mean death time for chick embryo (MDTE) was calculated. Then, as recommended by the latest version of the OIE Manual of Standards for Diagnostic Tests and Vaccines, for three strains (2008/Mali/ML007, 2009/Mali/ML008, 2007/Mali/ML029), the allantoic liquid was also inoculated in Specific Pathogen Free (SPF) one-day-old chicks to determine the intracerebral pathogenicity index (ICPI) [2].

### Amplification for Sequencing

Viral RNA was extracted from NDV-positive allantoic liquid using the Nucleospin RNA virus kit (Macherey Nagel) according to the manufacturer’s instructions. RNA was quantified by UV absorbance using a Nanodrop spectrophotometer (Thermo scientific). Complementary DNA was synthesized using 20 µl of eluted RNA with oligo pd(N)6 primer and the (M-MuLV) reverse transcriptase of the First-Strand cDNA Synthesis Kit (Ge Healthcare). Conventional PCR was carried out with 20 ng of cDNA using Taq DNA polymerase (Qiagen) according to the manufacturer’s instructions. Five distinct PCR runs allowed the amplification of the complete sequence of the F and HN genes. The primers used for amplification of F and HN genes are shown in Table 1. The PCR products were separated by gel electrophoresis using 2% w/v agarose gel in Tris-acetate buffer, stained with ethidium bromide, and visualized under ultraviolet light. The product was excised and purified using QiaQuick gel extraction Kit (Qiagen).

**Table 1:** Primers used for complete sequencing of the F and HN genes.

Primer	Sequence 5′-3′	Direction^a^	Nucleotide positionon AY741404	Gene target^b^	PCR product size	Reference
MFS1	GACCGCTGACCACGAGGTTA	F	4306	M	766	[30]
F3AS	TGCATCTTCCCAACTGCCAC	R	5072	F		c
F+4952	GCAGCCGCAGCTCTAATAC	F	4952	F	747	c
MFS3	GGCAATAACTGAGCCTTTGAG	R	5699	F		c
F+886	AATAATATGCGTGCCACCTA	F	5429	F	1040	c
HN49rev	GCGCCATGTRTTCTTTGC	R	6469	HN		c
P6A	ATCAGATGAGAGCCACTACA	F	6177	F	1120	[31]
HN886rev	ACTCCTGGGTAATTTGCCAC	R	7297	HN		c
3HNOV	GTCTTGCAGTGTGAGTGCAAC	F	7119	HN	1271	[30]
P7B	TCTGCCCTTTCAGGACCGGA	R	8390	L		[31]

a F, forward; R, reverse.

b M, F, HN, and L matrix, fusion protein, hemagglutinin-neuraminidase, and polymerase genes, respectively.

c primers designed on conserved sequences based on the alignment of the complete sequences of 219 F genes and 74 HN genes published in GenBank.

For F and HN genes, DNA was sequenced by the GATC-Biotech company using the same primers described for PCR reactions. For the full genome sequencing, genomic virus RNA was extracted from 200 µL of allantoic fluid using the QIAamp viral Rneasy Mini kit (QIAGEN, Hilden, Germany). RNAs were reverse transcribed using Superscript II (Invitrogen) with hexanucleotides. Twenty-six overlapping PCR products were obtained with Platinum Taq DNA polymerase (Platinum® TaqDNA polymerase High Fidelity, Invitrogen). All these primers were defined in the laboratory (primers available upon request). The DNA sequences were determined by sequencing, in both senses, using a Big dye Terminator v3.1 (Applied Biosystem) cycle sequencing kit according to the manufacturer’s instructions.

### Sequence Alignments, Phylogenetic Analyses, and Evolutionary Distances

The forward and reverse sequences were aligned using Vector NTI software version 10.3 (Invitrogen, Europe). The validated open reading frames of the different genes were aligned with others retrieved in GenBank using the CLUSTAL W program of the Mega5 software suite. The sequence of a class I NDV isolate was used as an outgroup. Full length genomes, including the genomes from the Malian and Madagascar strains, were aligned using Muscle, which is a faster and more reliable method than CLUSTAL W for larger sets of long sequences [32]. From the full genomes, individual and concatenated genes were generated using Mega5 software [33]. Putative recombination events in sequence alignment of available NDV genomes and the new Malian and Madagascar genomes studied here were investigated by RDP suite (versions RDP3 Alpha 44 and RDP4 Beta 4.16 [34]. This software uses multiple recombination signal detection methods like CHIMAERA, Maximum chi2, RDP, RECSCAN, and Geneconv. All recombined sequences were discarded before analysis. Phylogenetic reconstructions were first carried out by the Maximum Likelihood method implemented in Treefinder [35] (version of March 2011). The data sets consisted of the full genome sequences (13740 nt, n = 110), the six individual genes from these full genomes, the complete F genes (1653 nt, n = 741), the complete HN genes (1713 nt, n = 323), and the partial coding sequences (445 and 372 nt) of the F gene (for n = 796 and 1921, respectively). All these multiple sequence alignments are provided in Table S7. The best models of nucleotide substitution for each data set were selected from the uncorrected and corrected Akaike Information Criterion, the Hannan and Quinn performance-based decision theory [36], and Bayesian Information Criterion [37] of Treefinder version March 2010. A General Time Reversible (GTR) model with a discrete gamma distribution (+G) with 5 classes, allowing for invariant sites (+I), was the consensus substitution model proposed for the different data and was used for all Maximum Likelihood analyses. Bayesian inference was also performed with a GTR model using MrBayes_3.2.2 [38,(http://sourceforge.net/p/mrbayes/code/HEAD/tree/release/installers/)]. All Bayesian reconstructions were initially set at 100,000,000 trees with a sample frequency on the chains of 1/1,000 (targeted sample size = 100,000 trees). All priors were set by default, except the evolutionary model and the branch length. For the latter, an inverse gamma Dirichlet prior was selected to avoid overestimation of branch lengths by MrBayes [39], [40]. For all reconstructions, two runs were launched in parallel with two chains (one heated, one cold). A convergence rule between the two runs was set at a standard deviation of split frequencies lower than 0.01, which then stops the reconstruction. Alternatively, when the standard deviation of split frequencies followed a stationary fluctuation above 0.01 for several consecutive days, very long reconstructions were manually stopped. To assess convergence, the expected sampling sizes (ESS) for posterior probabilities and the Potential Scale Reduction Factor (PSRF) were checked for all reconstructions: validation criteria for all parameters were average ESS>200 and PSRF within [0.99 and 1.01] (as the chains converge in the runs, the variance between the runs becomes more similar and the PSRF approaches 1.0). The numbers of trees used for the generation of the consensus tree and values for the two convergence parameters are indicated in the results. Final trees were laid out using Figtree software, version 1.4.0. Topologies of the Maximum Likelihood and Bayesian consensus trees obtained for the different genes and the full genomes (n = 110 sequences in the different data sets) were compared by Treefinder using the Shimodaira and Hasegawa test [41]. The best representation was then selected. For the F gene, comparisons were performed using different phylogenetic methods, including Maximum Likelihood, Neighbor Joining, and Maximum Parsimony methods from Mega5 software [33] and the Bayesian approach for phylogenetic reconstruction. Branch support values were obtained using nonparametric bootstrapping with 1,000 resamplings for the first three phylogenetic methods, and the posterior probabilities for the Bayesian approach were estimated on 16,806 samples with a Burn-in phase for the first 25% of tree samples. The best tree for the F gene was determined by calculating the minimum branch length distance (K tree score) between the phylogenetic trees by the Ktreedist program [42]. The complete F gene data set was also used to calculate the mean evolutionary distances within and between clusters. The pairwise distance matrix was generated by Treefinder and analyzed in Excel. In addition to the evolutionary distance, nucleic and amino acid signatures specific for the different clusters were sought in the multiple alignments of the F gene and protein. New genotypes and sub-genotypes were assigned according to the criteria described by Diel et al [26] with the following modifications:

new genotypes and sub-genotypes were assigned on at least three independent isolates without a direct epidemiologic link in the phylogenetic trees generated for the complete F gene sequence and confirmed by at least two HN gene sequences, using both the Maximum Likelihood and Bayesian methods and the optimal nucleotide model (GTR +G +I, Г5), as determined by Treefinder.
the mean distance between genotypes and sub-genotypes, as determined by the distance matrix from the complete F gene sequence generated by the Maximum Composite Likelihood model of Treefinder, was higher than or equal to 0.100 and 0.03, respectively.
sub-genotypes were included into a monophyletic genotype and were identified by unique amino or nucleic acid signatures, as described in the results section.

## Results

### Detection and Initial Characterization of NDV Isolates in African Samples

Using real-time RT-PCR detection, we found 421 samples positive for NDV among 9,609 domestic samples (4.38%, 95% CI: 4–4.8%) and 211 positive samples among 10,343 collected from wild birds (2.04%, 95% CI: 1.8–2.3). Nine viruses were isolated from domestic birds in Mali, ten in Madagascar, and five in Ethiopia. However, note that eight of ten isolates from Madagascar were obtained from farms where clinical signs invoking Newcastle disease were obvious. This was not the case for the two other isolates from Madagascar and all the others from Mali and Ethiopia. In addition, two viruses were isolated from healthy wild birds in Madagascar. Unfortunately, no isolates were obtained from Mauritanian and Malian wild birds. However, 14 cleavage sites could be sequenced (Table 2). All isolates from Mali had a virulent cleavage site with at least four basic amino acids (sequences of domestic birds with an accession number in Table 2). Interestingly, the 2007/Mali/ML029 and 2007/Mali/ML031 isolates have five basic amino acids on their cleavage site and a V^118^ associated with the cleavage site (^112^RRRKR^116↓^FV), a motif described only rarely, and just recently in the neighboring country, Burkina Faso [22]. The second velogenic cleavage site (^112^RRQKR^116↓^FI) found in all other sequences from Malian domestic bird isolates was also encountered in some wild birds collected in Mali and Mauritania. This F cleavage site is identical to that of most NDV strains isolated in different parts of Asia and Africa [17], [19], [20], [21], [43]. The rest of the wild bird sequences from Mali and Mauritania shared a lentogenic F cleavage site (^112^GKQGR^116↓^LIA/V). All the strains isolated from domestic birds of Madagascar had five arginines (R) in the cleavage site ^112^GRRRRR^116↓^F and clustered in the same genotype XI as previously described [25]. The lentogenic cleavage site found in two wild birds of Madagascar was identical to the one in Mali and Mauritania (Table 2). The five Ethiopian isolates showed three velogenic cleavage sites: ^112^RRQKR^116^FV, ^112^RRRKR^116^FV, and ^112^RRHKR^116↓^FV. To our knowledge, a histidine in position 114 of a cleavage site of F protein has never been described before.

**Table 2:** Characteristics of viruses analyzed in this study.

Group	Sequence reference	Collecting year	Origin	Host	Cleavage site(aa 112–118)
domestic birds	2007/Mali/ML029/07	2007	Mopti market/Mali	*Gallus gallus*	RRRKR^↓^FV
	2007/Mali/ML038/07	2007	Mopti market/Mali	Guinea fowl	RRQKR^↓^FI
	2007/Mali/ML031/2007	2007	Mopti market/Mali	*Gallus gallus*	RRRKR^↓^FV
	2008/Mali/ML007/08	2008	Sikasso/Mali	*Gallus gallus*	RRQKR^↓^FI
	2008/Mali/ML225/08	2008	Mopti/Gninitogo/Mali	*Gallus gallus*	RRQKR^↓^FI
	2008/Mali/ML230/2008	2008	Mopti/Gninitogo/Mali	*Gallus gallus*	RRQKR^↓^FI
	2010/Mali/ML57071T	2010	Mali	*Gallus gallus*	RRQKR^↓^FI
	2010/Mali/ML57072T	2010	Mali	*Gallus gallus*	RRQKR^↓^FI
	2009/Mali/ML008*	2009	Mopti market/Mali	*Gallus gallus*	RRQKR^↓^FI
	2009/Madagascar/MGBBS	2009	Tsarahonenana/Antananarivo/Madagascar	*Gallus gallus*	RRRRR^↓^FV
	2010/Madagascar/MGF003C	2010	Amparihitsokatra/East_lakeside/Alaotra_region/Madagascar	*Gallus gallus*	RRRRR^↓^FV
	2011/Madagascar/MGF015C	2011	Ambohimandroso/West lakeside/Alaotra_region/Madagascar	*Gallus gallus*	RRRRR^↓^FV
	2010/Madagascar/MGF082T	2010	Amparafaravola/West_lakeside/Alaotra_region/Madagascar	*Gallus gallus*	RRRRR^↓^FV
	2011/Madagascar/MGF120T	2011	Anororo/West_lakeside/Alaotra_region/Madagascar	*Gallus gallus*	RRRRR^↓^FV
	2011/Madagascar/MGF166	2011	Anororo/West_lakeside/Alaotra_region/Madagascar	*Gallus gallus*	RRRRR^↓^FV
	2011/Madagascar/MGF192C	2011	Bejofo/East_lakeside/Alaotra_region/Madagascar	*Gallus gallus*	RRRRR^↓^FV
	2009/Madagascar/MGMNJ	2009	Manjakandriana/Antananarivo/Madagascar	*Gallus gallus*	RRRRR^↓^FV
	2011/Madagascar/MGS1130T	2011	Anororo/West_lakeside/Alaotra_region/Madagascar	*Gallus gallus*	RRRRR^↓^FV
	2011/Madagascar/MGS1595T	2011	Imerimandroso/East_lakeside/Alaotra_region/Madagascar	*Gallus gallus*	RRRRR^↓^FV
	2011/Ethiopia/ETH10065	2011	Dhera market/Ethiopia	*Gallus gallus*	RRQKR^↓^FV
	2011/Ethiopia/ETH10073	2011	Dire market/Ethiopia	*Gallus gallus*	RRHKR^↓^FV
	2011/Ethiopia/ETH8755	2011	Maliyu/Ethiopia	*Gallus gallus*	RRQKR^↓^FV
	2011/Ethiopia/ETHAN01	2011	Arsinegele/Ethiopia	*Gallus gallus*	RRQKR^↓^FV
	2011/Ethiopia/ETHMG1C	2011	Migra/Ethiopia	*Gallus gallus*	RRRKR^↓^FV
Wild birds	2009/Madagascar/MGA057C	2009	Alaotra_lake/Madagascar	*Anas Melleri*	GKQGR^↓^LI
	2009/Madagascar/MGA184C	2009	Alaotra_lake/Madagascar	*Anas erythrorhyncha*	GKQGR^↓^LI
	2008/Mali/DvML160_08	2008	Lake Fati/Mali	*Dendrocygna viduata*	RRQKR^↓^FI
	2008/Mali/NnML495_08	2008	Lake Horo/Mali	*Nycticorax nycticorax*	RRQKR^↓^FI
	2008/Mali/PpML283_08	2008	Lake Horo/Mali	*Porphyrio porphyrio*	GKQGR^↓^LI
	2008/Mali/CaMT197_08	2008	Banc d’Arguin/Mauritania	*Calidris alpina*	RRQKR^↓^FI
	2008/Mali/CpML476_08	2008	Lake Fati/Mali	*Charadrius pecuarius*	GKQGR^↓^LI
	2008/Mauritania/LgMTLG199_08	2008	Banc d’Arguin/Mauritania	*Larus genei*	RRQKR^↓^FI
	2008/Mauritania/LgMTLG223_08	2008	Banc d’Arguin/Mauritania	*Larus genei*	GKQGR^↓^LI
	2008/Mauritania/LgMTLG228_08	2008	Banc d’Arguin/Mauritania	*Larus genei*	GKQGR^↓^LI
	2008/Mauritania/LgMTLG232_08	2008	Banc d’Arguin/Mauritania	*Larus genei*	GKQGR^↓^LI
	2008/Mauritania/LgMTLG241_08	2008	Banc d’Arguin/Mauritania	*Larus genei*	RRQKR^↓^FI
	2008/Mauritania/LgMTLG243_08	2008	Banc d’Arguin/Mauritania	*Larus genei*	RRQKR^↓^FI
	2008/Mauritania/LgMTLG254_08	2008	Banc d’Arguin/Mauritania	*Larus genei*	RRQKR^↓^FI
	2008/Mauritania/LgMTLG258_08	2008	Banc d’Arguin/Mauritania	*Larus genei*	RRQKR^↓^FI
	2008/Mauritania/LgMTLG261_08	2008	Banc d’Arguin/Mauritania	*Larus genei*	RRQKR^↓^FI

**-** *full genome sequence.

The virulence of the 2007/Mali/ML029 isolate was verified by determining the MDTE and ICPI. The minimum dilution killing all inoculated eggs was 10^−8^ and the MDTE for this dilution was 48 and 60 hours in two independent tests, thus confirming the velogenic nature of this strain (mesogenic strain killed eggs after 60 hours). The ICPIs of 2007/Mali/ML029, 2008/Mali/ML007, and 2009/Mali/ML008 were all 1.8, close to the maximal score of 2. The ICPI of Madagascar isolates was previously established at 1.9 [25]. These results are in line with the virulent F cleavages site motifs described above.

### Genotyping of African NDV Isolates using Complete Sequences

To determine the genotype of the isolates, the F and HN genes of the African isolates were sequenced. The analyses of the total F and HN genes showed that 2007/Mali/ML031 and 2008/Mali/ML230 were 100% identical to 2007/Mali/ML029 and 2008/Mali/ML225, respectively (data not shown). For this reason, phylogenetic analyses were carried out on only seven of the Malian isolates (2007/Mali/ML029, 2007/Mali/ML038, 2008/Mali/ML225, 2008/Mali/ML007, 2009/Mali/ML008, 2010/Mali/ML57071T, and 2010/Mali/ML57072T). The degree of nucleic acid variation between these seven strains was 0.1–2.0% for the F gene (0.9–6.5% for the protein) and 2.0–2.3% for the HN gene (8.1–8.6% for the protein). Similarly, the divergences among the five Ethiopian strains were 0.2–1.0% and 0.1–0.7% for the F and HN genes, respectively (1.3–3.4% and 0.9–3.9% for the F and HN proteins, respectively). For the eight Madagascar isolates from domestic birds, the divergences ranged from 0–0.6% for the F gene (0–3.3% for the protein).

**Figure 1:**
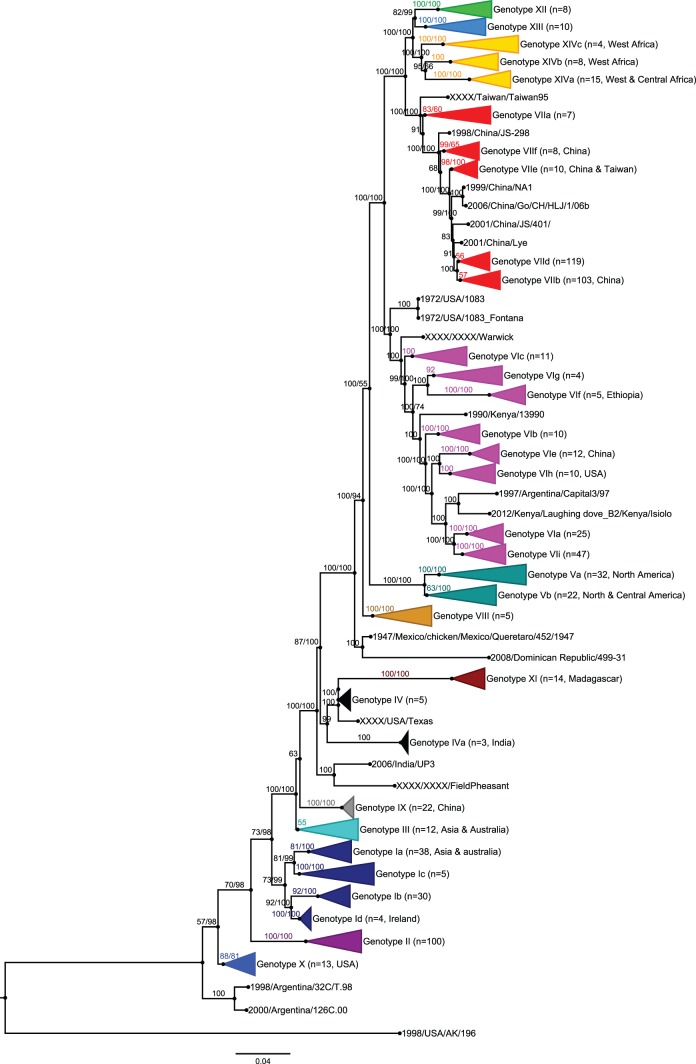
Phylogenetic analysis of 741 complete F nucleic acid sequences of NDV. Trees were constructed using Bayesian inference with 16,806,000 iterations and 1/1000 tree sampled in the chain. Minimal average ESS for all parameters was 1681, and PSRF were between 0.99996 and 1.00084. A class 1 virus sequence was introduced as an outgroup. Consensus tree posterior probabilities are indicated on the branch (first number). A Bayesian inference based on 323 complete HN nucleic acid sequences was also done (all details in Figure S3A, Figure S5J and Figure S9R). Since the F and HN trees were superimposable, only the F tree is represented; where informative, branch support values for HN are indicated after the F posterior probabilities. Sequences from this study are grouped in genotypes Ib (2 strains), VIf (5 strains), XI (10 strains), XIVa (1 strains), XIVb (5 strains), and XIVc (1 strain). The complete list of the 741 sequences, the corresponding multiple sequence alignments, and the tree in Newick and in Figtree format can be found in Table S8, Table S7, Figure S5G, and Figure S9O, respectively.

Phylogenetic analyses were performed by comparing the complete sequences of F and HN genes by the Maximum Likelihood and Bayesian inference methods with the best model of substitution proposed by Treefinder (GTR +G +I, Г5). The trees generated by the two methods were very close, as shown by the Shimodaira and Hasegawa test (Table S2). This was also the case for all individual genes and the full genome. However, the clustering of some strains was sometimes better with the Bayesian inference (data not shown). Bayesian trees were thus preferred for final representation. Since the topology of the two Bayesian trees rendered by the F and HN genes was identical, only the tree of the F gene is shown in Figure 1. The bootstrap values for 1000 replicates for the two genes are reported on this figure. Because sub-lineages 3a, 3b, 3c, and 3d described by Aldous et al. [1] were not found monophyletic in our reconstructions, a division into clusters gIII, gIV, gV, and gVIII and the use of nomenclature based on genotypes from Ballagi-Pordany et al. [12] and Lomniczi et al. [11] were preferred. For more clarity on NDV nomenclatures, the reader can see correspondences between various nomenclatures in Table S1. According to the criteria described in Material and Methods for the definition of genotype and sub-genotype and in Figure 1, 14 genotypes and 23 sub-genotypes are proposed here. The Malian isolates are all clustered with other isolates from West Africa [22], [23] into genotype XIV. This cluster is clearly separated from genotype VII in terms of genetic distances (0.139 versus 0.038, which is the average intra-genotype distance, see Table 3).The Malian isolates and others from Niger, Burkina Faso, Cameroon, Nigeria, Mauritania, and Ivory Coast also form three distinct sub-genotypes, here proposed as sub-genotypes XIVa, XIVb, and XIVc. The isolates from Madagascar domestic birds, all responsible for clinical outbreaks, were confirmed as members of genotype XI (Figure 1). Genotype VI contains highly diverse strains from pigeons and wild birds collected throughout the world. It is suspected that pigeon type-PMV1 emerged as a result of multiple transmissions of viruses from chickens to pigeons, but this hypothesis is currently not confirmed. Ujvári et al [44] revealed the existence of four sub-genotypes of VIb/1 PPMV1 and a new clade designated VIb/2 with recent isolates from Croatia and even more recent ones from Russia [45]. According to Diel’s criteria [26] and our analyses, four new sub-genotypes VI were defined. The Ethiopian isolates constitute the new sub-genotype VIf. Sub-genotype VIb/2 [45] including Russian strains was renamed sub-genotype VIg. Sub-genotypes VIh and VIi contain only isolates from the USA and isolates from pigeons and wild bird strains from the USA and Europe, respectively.

**Table 3:** Estimates of evolutionary distances between genotypes.

	I	II	III	IV	IX	V	VI	VII	VIII	X	XI	XII	XIII	XIV	XV
**I**	0.052														
**II**	0.140	0.018													
**III**	0.115	0.156	0.031												
**IV**	0.133	0.174	0.105	0.046											
**IX**	0.117	0.158	0.089	0.100	0.004										
**V**	0.237	0.277	0.209	0.188	0.204	0.068									
**VI**	0.226	0.266	0.199	0.177	0.193	0.195	0.077								
**VII**	0.225	0.263	0.197	0.176	0.192	0.194	0.158	0.036							
**VIII**	0.173	0.213	0.145	0.124	0.140	0.153	0.142	0.141	0.041						
**X**	0.124	0.129	0.140	0.158	0.142	0.262	0.252	0.250	0.198	0.021					
**XI**	0.235	0.275	0.207	0.160	0.202	0.289	0.279	0.278	0.225	0.260	0.015				
**XII**	0.232	0.272	0.205	0.184	0.200	0.201	0.165	0.128	0.148	0.258	0.285	0.021			
**XIII**	0.227	0.266	0.199	0.178	0.194	0.196	0.160	0.122	0.143	0.252	0.280	0.114	0.061		
**XIV**	0.244	0.283	0.216	0.195	0.211	0.213	0.177	0.139	0.160	0.269	0.296	0.134	0.128	0.086	
**XV**	0.135	0.175	0.108	0.086	0.102	0.129	0.118	0.117	0.064	0.161	0.188	0.124	0.119	0.136	0.000

Average intergenotype distance: 0.190.

Average intragenotype distance: 0.038.

The two Madagascar isolates from wild birds were classified as sub-genotype Ib. Beyond the new sequences of African isolates provided in this study, we confirm most of the genotypes/sub-genotypes proposed by Diel et al. [26], except sub-genotype VIIg and genotype XV, both including only recombined strains that were excluded from our data set to avoid any side-effect on the reconstruction. Relative to Diel et al [26], we propose the creation of sub-genotypes Ic, Id, IVa, VIf, XIVa, XIVb, and XIVc. Sub-genotype Ic (n = 5) is constituted by different strains from Europe, Asia and America. Sub-genotype Id (n = 4) is constituted by strains from Ireland only, and the genetic distance between this cluster and Ia, Ib sub-genotypes is 0.054 and 0.052, respectively. Our phylogenetic reconstructions show a cluster constituted by strains only from India (n = 3), with a common ancestor branched to genotype IV. These three viruses are distant enough (0.089) from genotype IV to propose a new sub-genotype IVa (Table 4). Genotype VI was previously classified into eight sub-genotypes by Wang et al, 2006 [46]. However, we confirm the existence of only four of these sub-genotypes, VIa, VIb, VIc, and VIe, in agreement with others [26]. Our five Ethiopian isolates are clustered in a distinct group branched to genotype VI. Its genetic distance from the other sub-genotypes VI (≥0.128), compared to the mean evolutionary distances between VIa, VIb, VIc, and VIe (<0.11, Table 4), supports the creation of a new sub-genotype, VIf. This sub-genotype was previously assigned to a cluster of Korean NDV strains described by Kwon et al. [47]. However, the phylogenetic tree was generated on a few isolates with shorter F gene sequences (319 nt) and by Neighbor Joining method. According to our reconstruction based on 796 sequences of 445 nucleotides (nt 55–500), the Korean isolates [47] clearly fall into sub-genotype VIc (Figure S3B, Figure S5K and Figure S9S). We also propose new sub-genotypes VIg, VIh, and VIi based on the phylogenetic sub-genotype distances observed between them (>0.03). Sub-genotype VIg was previously described by Wang et al, 2006 [46] to classify Chinese strains which, however, are included in VIc in our reconstructions. Genotype XIV, previously classified as sub-lineage 5f, 5g, and 5h by Snoeck et al. [22] and as a new lineage 7 by Cattoli et al [23], contains viruses obtained mainly in West and Central Africa between 2006 and 2008. The five strains from Mali isolated during this study are part of this cluster containing other strains from West Africa, previously described [23]. The genetic distance between this genotype and the two other close genotypes, XII and XIII, is very similar (0.134 to 0.128, Table 3), supporting the creation of a new genotype. In addition, three branches were identified within this genotype, and the genetic distance between these branches (>0.13) supports the creation of three new sub-genotypes: XIVa (former lineages 5g or 7b) that includes our strain 2008/Mali/ML007 and others from Burkina Faso, Niger, Nigeria, and Cameroon; XIVb (former lineage 7a) containing five of our Malian strains and others from Mauritania and Ivory Coast; and XIVc (former lineages 5f or 7d) containing our strain 2007/Mali/ML029 and another one from Niger.

**Table 4:** Estimates of evolutionary distances between sub-genotypes.

	Ia	Ib	Ic	Id	IVa	IV	Va	Vb	VIa	VIb	VIc	VIe	VIf	VIg	VIh	VIi	VIIa	VIIb	VIId	VIIe	VIIf	XIVa	XIVb	XIVc
**Ia**	0.016																							
**Ib**	0.078	0.019																						
**Ic**	0.070	0.094	0.053																					
**Id**	0.054	0.052	0.069	0.008																				
**IVa**					0.005	0.089																		
**IV**						0.014																		
**Va**							0.031																	
**Vb**							0.104	0.042																
**VIa**									0.021															
**VIb**									0.075	0.016														
**VIc**									0.105	0.076	0.043													
**VIe**									0.089	0.070	0.100	0.014												
**VIf**									0.158	0.128	0.138	0.152	0.023											
**VIg**									0.125	0.095	0.104	0.119	0.132	0.052										
**VIh**									0.081	0.062	0.092	0.063	0.145	0.111	0.031									
**VIi**									0.057	0.074	0.104	0.087	0.157	0.123	0.080	0.026								
**VIIa**																	0.052							
**VIIb**																	0.086	0.023						
**VIId**																	0.081	0.037	0.022					
**VIIe**																	0.075	0.041	0.036	0.018				
**VIIf**																	0.072	0.054	0.049	0.044	0.020			
**XIVa**																						0.021		
**XIVb**																						0.120	0.033	
**XIVc**																						0.142	0.137	0.062

Average intra-subgenotype distance: 0.03.

Genotypes XII and XIII were proposed recently by others [26] and are fully confirmed by our reconstruction including new sequences. Genotype XII contains virulent viruses recently isolated from poultry in South America and from geese in China [26]. Genotype XIII, previously classified as genotype VII [46], comprises virulent viruses that were isolated in Pakistan, Russia, Burundi, India, and Sweden between 1997 and 2008. Genotype VII, which comprises the largest number of NDV isolates and the highest diversity, is sub-divided into four sub-genotypes named VIIb, VIId, VIIe, and VIIf, as previously described [26]. Genotypes VII, XII, XIII, and XIV share a common ancestor. The recently described genotype XI found in Madagascar [25] was also consistently branched as a separate group from genotype IV.

Concerning sub-genotype IIa, we support on the basis of genetic distance from genotype II (0.13, Table 3) the proposal of Diel et al. [26] to introduce a new genotype X. Furthermore, the distinction between these two groups is reinforced by the exclusive combination of amino acid signatures (Table 5).

**Table 5:** List of specific signatures on the F complete gene for genotypes and subsequent sub-genotype discrimination.

Genotype	Exclusive signatures (amino acids/*nucleic acids*) Underline:specific signature to this cluster	Sub-genotype	Exclusive signatures (amino acids/*nucleic acids*) Underline: specific signature to this cluster
		Ia	–
			*T_1104_ T_1503_A_1539_*
		Ib	–
			*C_528_ A_753_ A_1029_ C_1542_*
I	V_17_ Q_422_	Ic	V_20_
	–		C*_1332_* T*_1377_*
		Id	–
			*G_132_ C_609_ A_1293_*
	V_19_ N_30_ L_69_ K_232_ I_386_ N_403_ K_421_ I_509_		
II	–		
III	T_16_ I_17_ V_26_ S_139_ Q_195_ T_288_		
	–		
IV	T_22_ R_115_	IVa	I_50_ S_132_ N_380_
	–		–
		Va	R_46_
V	A_106_ K_421_ S_476_ N_494_ A_508_ I_517_		–
		Vb	K_312_ V_516_
			–
		VIa	I_50_ S_132_ N_380_
			–
		VIb	T_19_ S_132_ S_406_
			–
	–	VIc	P_13_ A_132_ S_514_
VI	–	VIe	P_13_ I_50_ S_132_ S_515_
		VIf	S_132_ P_31_ E_342_
			**–**
		VIg	S_132_ H_272_ G_304_ I_537_
		VI h	T_9_ E_111_ S_132_ S_406_ V_432_
		VI i	I_50_ S_132_ N_380_K_494_
			*A_108_*
		VII a	V_255_ F_314_
		VIIb	V_52_ R_71_ I_255_ Y_314_ R_480_
			*T_1044_ G_1608_*
	S_176_	VIId	V_52_ I_255_ Y_314_
VII	–		*T_1044_ G_1608_*
		VIIe	V_52_ I_255_ Y_314_ R_480_
			*G_1608_*
		VIIf	A_29_ I_255_ Y_314_ R_480_
			–
VIII	T_11_ T_107_ A _203_ S_514_ V_516_		
	–		
IX	R_27_ N_30_ A_106_ R_115_ Q_195_ V_339_ I_386_ G_497_ I_509_		
	–		
X	G_4_ K_113_ G_115_ G_124_ Q_422_ Q_451_		
	–		
XI	L_18_ S_211_ S_272_ M_289_ L_384_ R_394_ H_411_ S_442_ I_522_		
	–		
XII	L_10_ I_43_ T_549_		
	–		
XIII	*A_192_ T_447_ A_696_ C_1033_ T_1368_*		
		XIVa	S_12_ P_15_ Y_27_ M_28_
			–
		XIVb	S_170_ N_550_
XIV	I_44_ K_51_		–
		XIVc	A_220_A_465_ L_510_
			–

The second column indicates the combination of specific amino (first line) and nucleic (second line) signatures that discriminate the genotypes. The fourth column shows the same for the sub-genotype discrimination and is applicable once the genotype has been assigned. Underlined amino or nucleic acids represent specific unique signatures.

Finally, with only two sequences from Dominican Republic and Mexico in our phylogenetic reconstructions, but showing a long branch between old and recent genotypes, the criteria were not fully met to assign a new genotype. However, very recent work adding more sequences from those countries supports the existence of a XVth genotype [27].

**Figure 2:**
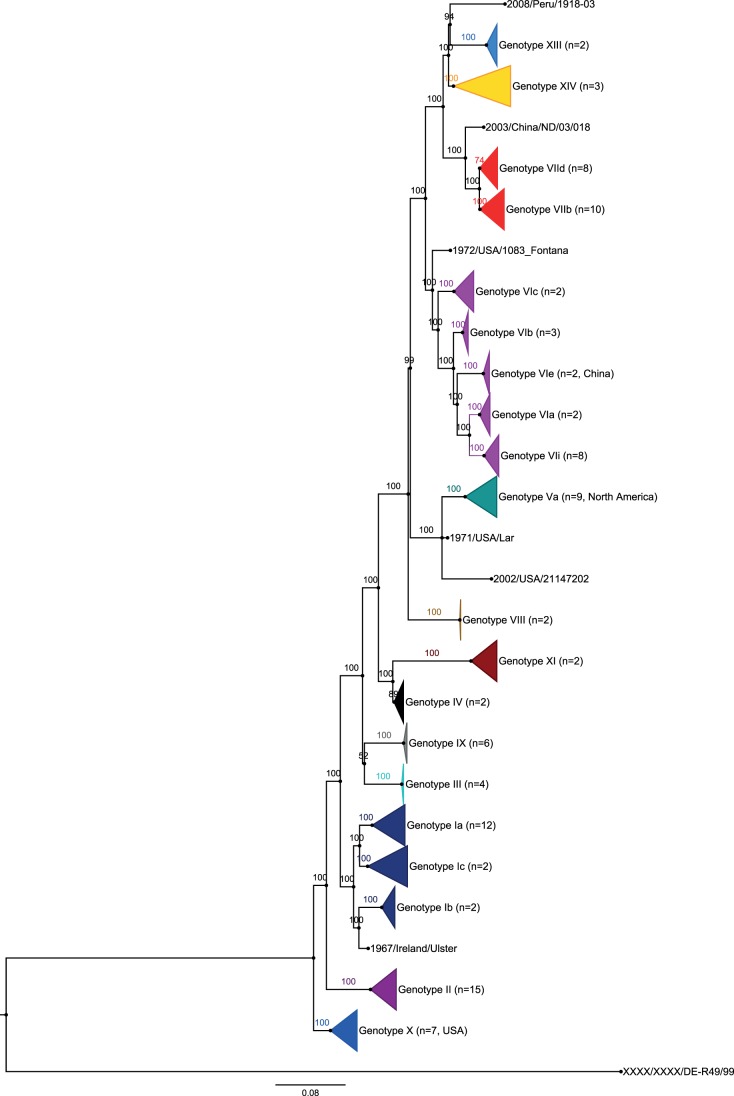
Phylogenetic analysis of 110 complete genome sequences. Trees were constructed using Bayesian inference with 2,960,000 iterations and 1/1000 tree sampled in the chain Minimal average ESS for all parameters was 365, and PSRF were between 0.99978 and 1.0059. A class I virus was used as an outgroup. Sequences from this study are three Malian strains, one in each of sub-genotypes XIVa, b, and c. The complete list of the 110 sequences, the corresponding multiple sequence alignments, and the tree in Newick and Figtree format can be found in Table S8, Table S7, Figure S5HandFigure S9P, respectively.

**Figure 3:**
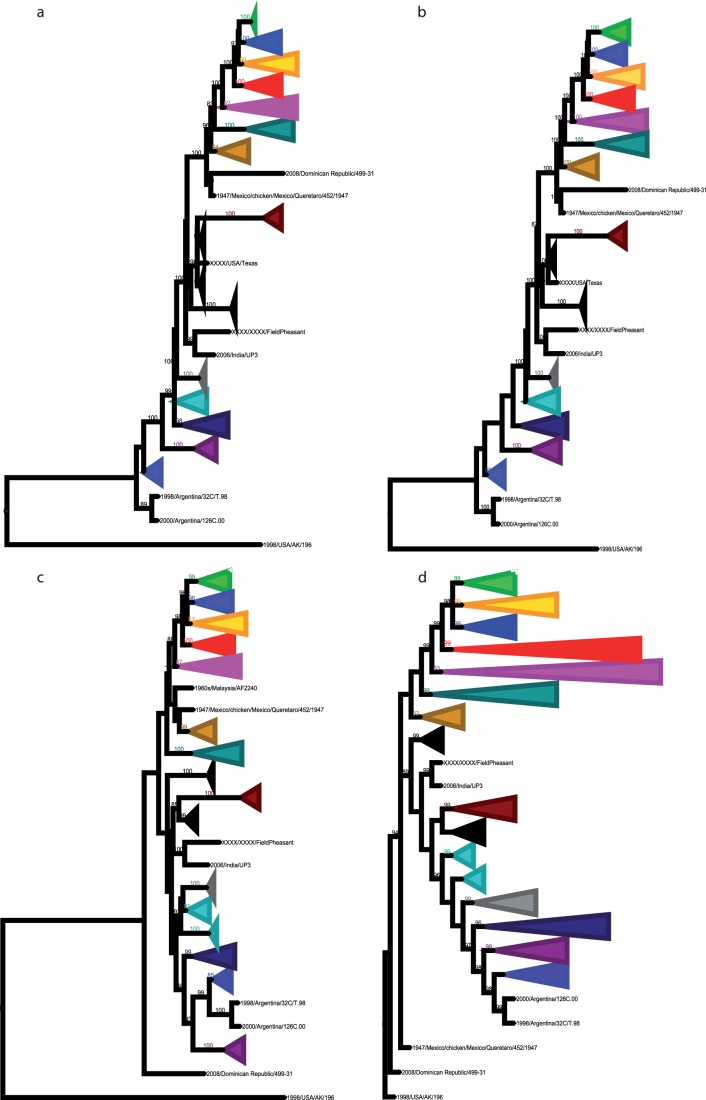
Comparison of tree topology after phylogenetic reconstruction on the 741 complete F gene sequences using four phylogenetic methods: (a) Maximum Likelihood (ML), (b) Bayesian (MrBayes), (c) Maximum Parsimony (MP), and (d) Neighbor Joining (NJ). Branch support values correspond to 1000 bootstrap replicates for ML, MP, and NJ, and posterior probabilities estimated for 10,000 samples of the Markov chain for MrBayes. The area of the triangle is proportional to the number of isolates within the genotype.

These results are confirmed by the identification of specific amino or nucleic acid signatures on the full F gene for all genotypes and sub-genotypes (Table 5). They are also confirmed by phylogenetic analyses based on the HN gene (Figure S3A), the N, P, M, and L genes (data not shown), and the full genomes (Figure 2). With the “Test topologies” option of Treefinder (Table S2), the most probable tree for representing the different sets of nucleic acid sequences was the one obtained with the full genome by Maximum Likelihood and Bayesian inference (tree in Figure 2). This tree branches the different genotypes in the following order from the root of the phylogenetic tree: X, II, I, III, (IV, XI), VIII, V, VI, and (VII, (XIII, XIV). The complete F tree confirms this classification, adding genotype IX between III and (IV, XI) and genotype XII with XIII and XIV. The complete HN tree switches genotypes III and IX but conserves the rest. Recombination analysis performed on all the sequences used in this study failed to identify any recombination events in our Malian, Ethiopian, and Madagascar isolates that could have interfered with the phylogenetic analyses. All our analyses were conducted using the Maximum Likelihood (ML) or Bayesian methods. To confirm our findings with these methods, a comparison with other methods was carried out on the F gene. The methods included Maximum Parsimony (MP) and Neighbor Joining (NJ) (JTT matrix with 5 categories for the gamma distribution) using Mega. The different representations are shown in Figure 3. The robustness of the trees is estimated by comparing bootstrap values for ML, NJ, and MP and the posterior probabilities of MrBayes for the main clusters (Figure 3)]. In addition, Ktreedist was used to compare the branch lengths between MrBayes, ML, and NJ (not applicable for MP). The best K-score was obtained with the Bayesian inference (Table S4).

### Confirmation of the New Nomenclature using Shorter Sequences

**Figure 4:**
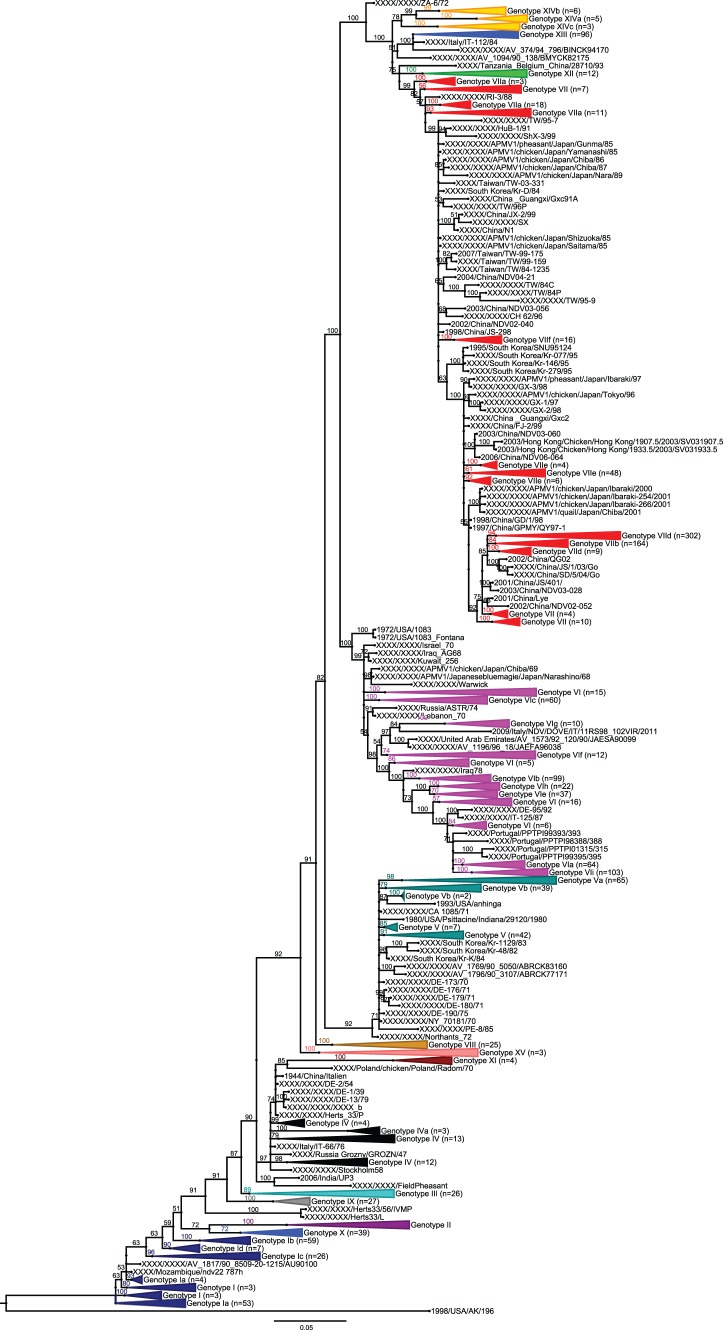
Phylogenetic analysis of 1921 partial F gene sequences based on nucleotides from positions 1 to 372. Trees were constructed using Bayesian inference with 47,408,000 iterations and 1/1000 tree sampled in the chain. Minimal average ESS for all parameters was 88, and PSRF were between 1.00003 and 1.01335. A class I virus was used as an outgroup. Sequences from this study are 5 Malian strains (1 in sub-genotype XIVa, 3 in XIVb, and 1 in XIVc), 5 Ethiopian strains (VIf), and 4 Madagascar strains (XI). The complete list of the 1921 sequences, the corresponding multiple sequence alignments, and the tree in Newick and Figtree format can be found in Table S8, Table S7, Figure S5I, and Figure S9Q, respectively.

Since partial F gene sequences are often used in the literature, we made a Bayesian phylogenetic reconstruction with 1921 sequences of 372 bases retrieved in GenBank. The genotypes were generally discriminated as with the full F gene except for genotype I, which cannot be maintained as monophylogenetic because its sub-genotypes are spread around genotypes II and X (Figure 4). At the sub-genotype level, the partial sequence does not allow precise delineation and so it is complicated to identify strains that are not close enough to the standard sequence of a given sub-genotype. The reconstruction with partial F gene is therefore less accurate than with the full F gene as shown by the inability to achieve the convergence rule and validation criteria with MrBayes (standard deviation of split frequencies lower than 0.01 or minimal average ESS <200 and PSRF within [0.99 and 1.01], see Figure 4 caption). It suggests that a 372-base-long segment should be the minimal genetic information required for correct genotyping but should be used with caution for further sub-clustering.

## Discussion

In this study, a comprehensive phylogenetic reconstruction of NDV strains was carried out using robust inference methods based on Maximum Likelihood and MrBayes. We included sequences in public databases representing all genotypes described so far and new isolates obtained and sequenced in this study in the framework of an international survey on wild and domestic birds in Africa. In this active surveillance survey, the prevalence of NDV in domestic birds was three times lower than that found in a study carried out in Burkina Faso [48]. In that study, the Burkina samples were also tested for the presence of influenza A viruses; the prevalence was estimated to be 3.2%. This prevalence was also three times higher than ours (data not shown). It is unknown whether this reflects a difference in sensitivity of detection methods used by the investigating groups or in the virus prevalence in different African countries. The presence of NDV in apparently healthy poultry in Africa is not surprising with regard to low or moderate virulent strains. However, most of our isolates have a cleavage site of virulent strains. In addition, some representative isolates were confirmed to be highly virulent in experimental challenges under controlled conditions. It remains unclear whether the apparent absence of signs in poultry infected by virulent strains results from the incubation time before disease onset or an incomplete vaccine protection (no information available about the pre-immune status). In line with the second assumption, the fact that viruses currently involved in Mali and Ethiopia belong to new genotypes (XIV and VIf, respectively) could question the efficacy of vaccines used in these countries, which all derived a long time ago from genotype II.

The data sets used in this study and the comparison of different methods for tree reconstruction allowed us to refine the NDV classification. The clustering of the different genotypes as well as the analyses of the minimal distances between genotypes and the identification of specific signatures for each sub-genotype show that the nomenclature proposed by Ballagi-Pordany [12] and Lomniczi et al [11] is more appropriate to describe the phylogenetic relationships between the isolates than the lineage nomenclature proposed by Aldous et al [1] and adopted by others [11], [12], [13], [26].

Most NDV phylogenetic reconstructions are based on the most variable part of the F gene (nucleotide 47 to 420). However, recombination occurs in NDV genomes and thus the use of only a partial sequence of the F gene for genotyping can lead to incomplete or false conclusions [49]. For instance, Qin et al. observed that strain SRZ03 isolated in China was the result of a recombination inside the F open reading frame between a genotype II and a genotype VII strain [49]. Moreover, Chong et al. showed incongruence in genotyping for 6 out of 54 complete genomes of NDV because of recombination events [50]. Here, to avoid any mistake due to recombination events, an analysis was carried out to show the absence of recombinations. We found evidence of recombination in 47 strains in the public database (Table S6). More than 45% of the recombinations of F gene involved the most variable part of the gene, thus including the region habitually used for NDV phylogeny. In this study, all phylogeny is based on the analysis of the complete sequence of the F and HN genes and confirmed by full genomes and N, P, M, and L genes (data not shown). In addition, different methods have been used for NDV phylogenetic analysis, like Neighbor Joining (NJ) [4], [11], [12], [13], [49], Maximum Likelihood (ML) [1], [50], [51], or Maximum Parsimony (MP) [44]. Using different algorithms for phylogenetic reconstructions may have an impact on the interpretation of the trees and thus on NDV classification. To strengthen our observations and interpretations, we compared the performances of four main methods (NJ, ML, MP, and Bayesian) for phylogenetic tree reconstruction on the complete F gene sequence. The robustness of the trees was assessed by comparison of the bootstrap values for NJ, MP, and ML and posterior probabilities of the Bayesian approach [38]. A K-score analysis was also done to compare MrB, ML, and NJ using the Ktreedist program [42]. From these comparisons, the performance of MrBayes was slightly better than ML, and both were clearly higher than NJ and MP. In our previous publication describing new genotype XI in Madagascar [25], we discussed some clustering incongruence between phylogenetic trees based on full genome and on the 374 nt fragment of the F gene. At that time, our analyses were carried out using nucleotide sequences and NJ method. The robustness of the results found in the current study in comparison with our previous ones reinforces the concept that phylogenetic analyses based on nucleic acid sequences and Maximum Likelihood or Bayesian approaches are more trustworthy.

Our results confirm most of the genotypes/sub-genotypes proposed by Diel et al, [26], except sub-genotype VIIg and genotype XV, which we do not recommend for validation at the moment. Both include only recombined strains and there is no clear indication that all strains in each of these clusters are phylogenetically related (e.g. arising from a common ancestor). Indeed, there is no consistency in the position of recombination events in the F gene and in the strains that are involved in these events, for both genotypes VIIg and XV, making it difficult to assume a monophyletic lineage for each of these clusters. We describe then the existence of 14 genotypes and propose 10 new sub-genotypes: Ic, Id, IVa, VIf, VIg, Vh, VIi, XIVa, XIVb, and XIVc. Furthermore, all the clusters have exclusive single or combined amino/nucleic acid signatures. Genotype VI was previously classified into eight sub-genotypes by Wang et al [46]. We confirm only four of these sub-genotypes (VIa, b, c, and e) but add four additional clusters consisting of Ethiopian strains grouped in a new sub-genotype VIf, Russian strains grouped in sub-genotype VIg (previously described as VIb/2), and North American strains clustered in sub-genotype VIh and sub-genotype VIi.

All seven strains isolated from Mali in this study clustered in genotype XIV with other strains previously described by Snoeck et al. [22] and Cattoli et al. [23]. All strains in genotype XIV have a cleavage site characteristic of virulent strains. It contains at least three basic amino acid residues (arginine or lysine) at the cleavage site of F protein (positions 113 to 116) in addition to a phenylalanine residue at position 117 [2]. The three distinct branches within this genotype with a high degree of genetic variability between them and the specific amino acid signatures allow sub-clustering into XIVa, b, and c. The analysis of recent western African isolates suggests that these viruses have evolved from a common ancestor with genotype VII, which contains a majority of strains from Asia [22], [23]. The ancestor seems to have generated two distinct lineages: on the one hand, genotype VII and on the other hand, genotypes XII, XIII, and XIV. The fact that only African strains are found in genotype XIV is in favor of the hypothesis that a unique variant was introduced some time ago, possibly from Asia, and that it subsequently evolved into different sub-genotypes.

In the framework of this study, we have also detected eight new virulent NDV strains in Madagascar, all of them clustered in genotype XI previously described by our group [25]. Moreover, we consolidate the identity of genotype XI by nine exclusive amino acid signatures. The presence of only genotype XI strains in NDV outbreaks in domestic birds and the PCR detection of such strains in healthy wild birds (data not shown) suggest large circulation of this particularly virulent genotype between domestic and wild bird compartments in Madagascar.

For NDV tree reconstruction and genotyping, we suggest using a Bayesian or Maximum Likelihood inference on the complete F gene, as recombinations may occur. In this study, the genotypes were generally well discriminated with the full F gene. Consistent reconstruction cannot be performed with full confidence with sequences of only 375 bases of F gene, as is usually done. However, phylogenetic trees based on 375-nt can at least discriminate major genotype clusters, even if discrimination between genotypes I and II and certain sub-genotypes may become less reliable. In large epidemiological surveys, like the one that allowed us to identify new (sub-) genotypes in West Africa, Ethiopia, and Madagascar [this study, 25], it is not always possible to generate the full-length sequence of the F gene from PCR positive swabs, in particular when all attempts to isolate the virus failed. In these situations, a phylogeny based on partial F gene sequences may help but the limitations of such an approach should be kept in mind.

## Supporting Information

Table S1 **Table of correspondence between the nomenclature by Aldous** ***et al.*** **** [**1**] **or Lomniczi** ***et al.*** **** [**11**] **and the new nomenclature proposed for all genotypes based on our results and those of Diel et al.** [**26**] **and Courtney et al.** [**27**] **.**
(DOCX)

Table S2 **Comparison of tree topologies generated on the individual gene and the full genome sequence data set (110 sequences in the different data sets) by Maximum Likelihood and Bayesian inference.** Comparison was made by Treefinder using the Shimodaira and Hasegawa test [41].
(XLSX)

Figure S3 **Supplementary tree representations.** Figure A: Phylogenetic analysis of 323 complete HN nucleic acid sequences of NDV. Trees were constructed using Bayesian inference with 2,648,000 iterations 1/1000 tree sampled in the chain. Minimal average ESS for all parameters was 400, and PSRF were between 0.99976 and 1.00196. A class 1 virus sequence was introduced as an outgroup. Consensus tree posterior probabilities are indicated on the branch. Sequences from this study are grouped in genotypes VIf (5 Ethiopian strains), XI (8 Madagascar strains), XIVa (1 Malian strain), XIVb (5 Malian strains), and XIVc (1 Malian strain). The complete list of the 323 sequences, the corresponding multiple sequence alignments, and the tree in Newick and Figtree format can be found in Table S8, Table S7, Figure S5J. and Figure S9R, respectively. Figure B: Phylogenic inference of 496 partial F gene sequences (445 nt) using MrBayes. Trees were constructed using Bayesian inference with 6,013,000 iterations and 1/1000 tree sampled in the chain. Minimal average ESS for all parameters was 614, and PSRF were between 0.99989 and 1.00057. A class I virus was used as an outgroup. Sequences from our study are 5 Malian strains (1 in sub-genotype XIVa, 3 in XIVb, and 1 in XIVc), 5 Ethiopian strains (VIf), and 12 Madagascar strains (2 and 10 in genotypes Ib and XI, respectively). The complete list of the 1921 sequences, the corresponding multiple sequence alignments, and the tree in Newick and Figtree format can be found in Table S8, Table S7, Figure S5I,and Figure S9Q, respectively.
(ZIP)

Table S4 **Comparison of trees generated by MrBayes, Maximum Likelihood, and Neighbor Joining on 741 F gene sequences.** The comparison was made by calculating the minimum branch length distance (K tree score) between the phylogenetic trees using the Ktreedist program [42]. The minimal score represents the best tree.
(XLSX)

Figure S5 **Trees generated by MrBayes in Newick format for 741 F gene sequences (Figure G), 110 full genome (Figure H), 1921 F partial sequences –372** **nt (Figure I), 796 F partial sequences –445** **nt (Figure N) and 323** **HN sequences (Figure J).** Trees generated in Newick format on F gene sequences by Maximum Likelihood (Figure K), Neighbor Joining (Figure L) and Maximum Parsimony (Figure M).
(ZIP)

Table S6 **Detection of recombination events in the different sequence data sets by RDP suite.**
(XLSX)

Table S7 **Multiple sequence alignments in Fasta format of 741** **F gene sequences, 110 full genome, 1921** **F partial sequences –372** **nt, 796** **F partial sequences – 445** **nt and 323** **HN sequences.**
(ZIP)

Table S8 **List of sequences included in the different data sets (full genome, complete F gene, partial F gene –372 nt, partial F gene –445 nt and complete HN gene).**
(XLSX)

Figure S9 **Trees generated by MrBayes in Figtree format for 741** **F gene sequences (Figure O), 110 full genome (Figure P), 1921** **F partial sequences –372** **nt (Figure Q), 323** **HN sequences (Figure R) and 796** **F partial sequences –445** **nt (Figure S).**
(ZIP)
