# Supplementary material for: New Avian Paramyxoviruses Type I Strains Identified in Africa Provide New Outcomes for Phylogeny Reconstruction and Genotype Classification
Source: PLoS One. 2013 Oct 18;8(10):e76413. doi: 10.1371/journal.pone.0076413 (PMC3799739; doi:10.1371/journal.pone.0076413)
Supplement: Table S1 — Table of correspondence between the nomenclature by Aldous et al. [1] or Lomniczi et al. [11] and the new nomenclature proposed for all genotypes based on our results and those of Diel et al. [26] and Courtney et al. [27] . (DOCX) [file pone.0076413.s001.docx]

S1 : Table of ccorrespondence between ancient nomenclature by Aldous *et al.* or Lomniczi *et al.* [11] completed by Kwon et al [48] and the new proposed nomenclature for all genotypes based on our results and those of Diel *et al.* [26] and Courtney *et al.* [27].

| Ancient nomenclature  (Lomniczi, Kwon) | Ancient nomenclature (Aldous) | Proposed nomenclature |
| --- | --- | --- |
| - | Lineage 6 | Class 1 |
| Genotype I | Lineage 1 | Genotype Ia |
| Genotype I | Lineage 1 | Genotype Ib |
| Genotype I | Lineage 1 | Genotype Ic |
| Genotype I | Lineage 1 | Genotype Id |
| Genotype II | Lineage 2 | Genotype II |
| Genotype II |  |  |
| Genotype III | Lineage 3a | Genotype III |
| Genotype IV | Lineage 3b | Genotype IVa |
| Genotype IV | Lineage 3b | Genotype IV |
| Genotype V | Lineage 3c | Genotype Va |
|  | Lineage 3c | Genotype Vb |
| NA | Lineage 4a | Genotype VIa |
| Genotype VIb | Lineage 4b | Genotype VIb |
| Genotype VIc | Lineage 4c | Genotype VIc |
| Genotype VIe | Lineage 4b | Genotype VIe |
| Genotype VIf | NA | Genotype VIc |
| NA | NA | Genotype VIf |
| Genotype VII | Lineage 5e | Genotype VII |
| Genotype VII | Lineage 5c | Genotype VII **?** |
| NA | Lineage 5d | Genotype VIId |
| NA | Lineage 5d | Genotype VIIe |
| **NA** | Lineage 5e | Genotype VIIf |
| NA | Lineage 3d | Genotype VIII |
| NA | Lineage 3a | Genotype IX |
| NA | Lineage 2 | Genotype X |
| NA | NA | Genotype XI |
| NA | Lineage 5b | Genotype XII |
| NA | Lineage 5b | Genotype XIII |
| NA | Lineage 5g*** or 7b* | Genotype XIVa** |
| NA | Lineage 7a* | Genotype XIVb |
| NA | Lineage 5f*** or 7d* | Genotype XIVc |

* proposed by Catolli et al. [23]

** previously proposed by our team as subgenotype VIIi [24]

*** proposed by Snoeck et al. [22]

NA: not applicable
